# Dental Treatment under General Anesthesia in Patients with Special Needs Provided by Private and Public Healthcare Services—A Retrospective, Comparative Study

**DOI:** 10.3390/healthcare10061147

**Published:** 2022-06-20

**Authors:** Marcello Alves Marinho, Flávia Cristina Teixeira Ramos, Andréa Lanzillotti Cardoso, Geraldo Oliveira Silva-Junior, Marcelo Daniel Brito Faria, Luciana Freitas Bastos, Arkadiusz Dziedzic, Bruna Lavinas Sayed Picciani

**Affiliations:** 1 Graduate Program in Dentistry, Health Institute of Nova Friburgo, Fluminense Federal University, Friburgo 20950-000, RJ, Brazil; marinhomarcello91@gmail.com (M.A.M.); andrealanzi.ppc@gmail.com (A.L.C.); silvajuniorgo@gmail.com (G.O.S.-J.); 2 Center of Dental Radiology and Care to Patients with Special Needs, Piquet Carneiro Polyclinic, Rio de Janeiro State University, Rio de Janiero 20950-000, RJ, Brazil; flaviatramos@live.com (F.C.T.R.); mdanbf@yahoo.com (M.D.B.F.); lucianafreitasbastos@yahoo.com (L.F.B.); 3 Department of Preventive and Community Dentistry, School of Dentistry, Rio de Janeiro State University, Rio de Janiero 20551-030, RJ, Brazil; 4 Department of Diagnosis and 2Therapeutics, School of Dentistry, Rio de Janeiro State University, Rio de Janiero 20551-030, RJ, Brazil; 5 Department of Conservative Dentistry with Endodontics, Medical University of Silesia, 40-551 Katowice, Poland

**Keywords:** dental general anesthesia, special needs, disabled persons, retrospective study

## Abstract

In special care dentistry, general anesthesia (GA) is considered as an alternative option to facilitate treatment for uncooperative patients with special needs (PSN) who require invasive dental interventions. Objective: to evaluate the profile of dental treatment procedures performed and the characteristics of PSN who underwent dental treatment under GA, provided by private and public healthcare providers. Methods: A retrospective, observational study involving a sample of 100 PSN treated in hospital and specialist secondary care settings. Demographic data and clinical information were collected. The analysis of data was performed using descriptive analysis and frequency statistical tests. Results: out of 100 participants, 63% of the PSN who received care in the private sector and the remaining 37% of PSN registered with public-funded care providers, aged 6 to 80 years old, were treated under GA. Autistic spectrum disorder was the most common medical diagnosis recorded (33%). More than half (52%) of the PSN treated by private care providers sought specialist care in an outpatient setting prior to GA vs. 5% of the PSN treated by public-funded providers. The utilization of sedation prior to GA was more common in the private sector. A vast majority (86%) of all subjects underwent multiple dental extractions (86%) and restorations (62%). Conclusions: comprehensive dental care under GA, which composes an integral part of special care dentistry, can be safely provided in a hospital setting, in both private and public sectors. While early intervention using sedation and behavioral management partially mitigates the need for dental care under GA, the vast majority of PSN may require dental treatment under GA in future to facilitate complex dental care.

## 1. Introduction

Persons with special needs (PSN) are characterized by a wide spectrum of long-term physical, mental, intellectual, or sensory impairments, affecting their daily activities and influencing the provision of healthcare, including dental care [1,2,3,4]. In the field of special care dentistry, this group of vulnerable individuals manifests a generally higher rate of oral diseases, such as caries and periodontal diseases, and often reveals substantial additional needs of extensive, multidisciplinary, and complex dental treatment [5,6]. In a considerable proportion of PSN, a ‘gentle approach’, behavioral management, and/or cognitive behavioral therapy is deemed ineffective, and such patients require pharmacological management in the form of conscious sedation utilizing several pathways, as well as dental general anesthesia (GA) in the most severe cases [7,8,9,10]. GA should be considered as an alternative of last resort for PSN when no other feasible and safe option for controlling their behavior is available. Treatment under GA applied for dental procedures can be clinically justified as the first treatment option for PSN who are not compliant and uncooperative due to severe systemic diseases, where there is a need for urgent care due to pain and/or in a complex dental treatment course. Hence, definite diagnosis and strict radical treatment planning are essential elements [11,12,13,14]. The family members or persons with legal responsibility/power of attorney must understand GA advantages, risks involved, and consequently be able to provide informed valid consent for dental treatment under GA in the best interest of patients [2,11,12,13,14]. The GA benefits include comfort and convenience for the patient as well as the operator and predictable and efficient patient management, as well as safety factors due to strictly controlled protocol, including a secure hospital environment with direct access to critical care facilities considering medical emergency episodes [12,13]. On the other hand, the potential risk of systemic complications is deemed higher compared with conscious sedation techniques. Hence, a thorough, structured anesthesia risk assessment constitutes a paramount element of GA referrals and pre-GA evaluation. This process includes several stages during assessment of co-morbidities and general health status, medications, drugs interactions, allergies, body mass index, airways assessment, and history of previous GA episodes.

Globally, the vast majority of dental care under GA is provided by public healthcare services, particularly for the most complex clinical cases in patients with severe comorbidities. The range of GA techniques and GA agents is utilized for dental patients, depending on age, clinical indications, providers’ facilities, health status, and procedures involved. Despite reasonably uniform GA protocol, there are significant discrepancies in protocols/guidance for dental treatment under GA between countries and regions worldwide, regardless of its public vs. private profile. Medico-legal, consent, financial, and competency considerations play an important role in setting up multidisciplinary teams providing routine and urgent dental care under GA, particularly for underserved, vulnerable, and special needs populations. Whist private sector healthcare providers support dental care delivery for PSN, the substantial cost of a GA session associated with facilities, equipment, and professional teams restricts the direct access to GA in less advanced and developing countries. Undoubtedly, private care sector services contribute to a provision of sedation and pharmacological management of, e.g., anxious patients, and their involvement in dental treatment under GA is considered as not substantial, with a wide variation between continents. Contrarily, public providers are involved in dental sedation to a lesser extent, which may impact long-term treatment outcomes. In some instances, the same anesthetist team may be involved in GA provision in different services (public and private) across regions due to a shortage of qualified personnel and/or healthcare system network organization.

Although GA is deemed a safe, routine medical procedure, intra- and post-operative complications may occur as a result of human error or factors associated with a patient’s condition [14,15,16]. Therefore, a thorough pre-GA risk assessment is deemed a crucial element of GA protocol. It has to be emphasized that, despite its benefits, GA is regarded as an economical and financial burden for care providers because it requires a specific hospital infrastructure, compatible with the complexity of planned procedures, specialist support of anesthesiologists, and interdisciplinary team to provide safe and efficient care [15,16]. Detailed information related to the treatment of PSN under GA is scarce, despite the well-recognized benefits [3,4,5,6,7,8,9,10,11,12,13,14,15,16]. The general characteristics and health profile of patients subjected to dental procedures under GA are essential when planning a session under GA and indicating this intervention, as well as composing an integral element of public health reporting data to facilitate appropriate strategic planning for improving a healthcare provision.

The study was aimed to evaluate the main characteristics and treatment profile of patients who require special care who are subjected to dental treatment under general anesthesia provided by public and private healthcare providers. The comparison of previous attempts of sedation and non-pharmacological management offered by these providers was an additional objective of the study.

## 2. Materials and Methods

### 2.1. Sample Selection

The structured retrospective, observational study was executed with the use of an adequately calculated sample size, including selected medical records of PSN referred for dental treatment under GA to the public dental center for disabled patients of the Policlínica Piquet Carneiro at the Universidade do Estado do Rio de Janeiro (UERJ), as well as to the private clinic. The assessment of medical records involved patients treated under GA between January 2016 and December 2020. Both services formulate the reference level of secondary and tertiary care in Brazil, comprising various dental/medical specialists dealing with PSN. These patients were under care of the public service in the surgical center available on site, whereas GA procedures were performed at private hospitals in the region in case of PSN treated by the private providers. The exclusion criteria comprised lack of essential data in patients’ records, borderline cases of individuals who did not meet fully PSN criteria, and ‘shared service’ cases when patient was treated by both providers. There was an age limit set at the age of minimum 3 years old.

Demographic data comprised the following information: gender (male or female), ethnicity (white or other), age (considering only full years completed at the time of general anesthesia), and city of residence (the municipality of Rio de Janeiro or other). The clinical data provided included: medical diagnosis/primary condition, disability status according to the International Association of Disability and Oral Health (IADH), primary dental complaint as reported by patient or legal guardian, care-seeking history prior to GA, need for new dental interventions under GA, and specific dental procedures carried out under GA. The ASA status was recorded for each patient, including basic medical comorbidities and medications. The study was conducted in accordance with the Declaration of Helsinki and approved by the Institutional Ethics Committee under CAAE register number 24279314100005259/2014.

### 2.2. Retrospective Study Protocol

All patients were assessed by dental practitioners/specialists with special interest in special care dentistry dealing with PSN during initial consultation in order to determine whether they had to undergo additional care prior to GA and to establish whether any previous dental care was completed under sedation or with support of behavioral management techniques alone. To optimize the interpretation, the dental procedures performed, dental extractions and restorations (regardless of the material used), were categorized using 3-grade scale: (1) no procedure, (2) 1 to 4 procedures, and (3) more than 5 procedures completed overall. The following procedures were divided into ‘yes’ or ‘no’ categories: (a) non-surgical debridement (periodontal professional plaque removal), (b) dental prophylaxis and polishing, (c) topical application of fluoride varnish, (d) endodontic treatment, (e) prosthetic work (crowns/dentures), (f) surgical lengthening of clinical crown, and (g) biopsy of oral tissue/lesion for oral medicine investigation.

### 2.3. Statistical Analysis

The data were extracted into Microsoft^®^ Excel for Mac, version 16.49 (US, Cal). A descriptive analysis of the studied variables was performed via proportions for categorical variables and using means, standard deviations, minimum–maximum values, mode, and medians for numerical variables. Statistical tests were executed using the Statistical Package for the Social Sciences^®^, version 22.0 (SPSS) software. Pearson’s chi-squared test was used to evaluate the difference between categorical variables, whereas the analysis of variance with one factor (one-way ANOVA) was used for numerical variables. Statistical significance for all analyses was 5% (*p* < 0.05).

## 3. Results

The retrospective study group comprised 100 participants meeting the criteria of PSN, subjected to GA that was clinically indicated and utilized for dental treatment. Valid, informed consent was obtained from the patient (capacity competent) or legal guardian. A vast majority of the PSN (63%) have been receiving dental care from private providers, in comparison to 37% registered with public services (Table 1). Healthcare providers and anesthetic teams in both sectors used a similar standard GA technique, including intravenous induction, nasotracheal intubation, initial inhalation anesthetic, and maintenance with mainly sevoflurane anesthetic gas. The average duration time of the GA session was three hours. No significant/severe side effect of GA or GA-related complications were recorded, and the full recovery of all patients was uneventful.

### 3.1. Study Group Characteristics and Demographics

In total, 62% patients were male, whereas 38% were female. The white ethnicity background group represented 58% of the PSN and was dominant in the private healthcare group (*p* = 0.035). Considering the entire sample, the age ranged from 6 to 80 years, with a mean age of 30 years (DM = 17) in the private sector group and 28 years (DM = 11) in the public sector group. A vast majority of patients came from urban areas (Table 1).

The most diagnosed medical condition was autistic spectrum disorder (33%), followed by intellectual disability (27%) and cerebral palsy (10%) (Table 2), whereas the most prevalent IADH categories were behavioral and psychiatric disorders (41%) and mental as well as physical disabilities (39%) (Table 3). PSN treated by public providers exhibited more complex medical conditions and compromised health status, with a higher GA-related risk as demonstrated based on ASA preoperative risk assessment (Table 4). Healthcare providers and anesthetic teams in both sectors used a similar standard GA technique, including intravenous induction, nasotracheal intubation, initial inhalation anesthetic, and maintenance with mainly sevoflurane anesthetic gas. The average duration time of the GA session was three hours. No significant/severe side effect of GA or GA-related complications were recorded, and the full recovery of all patients was uneventful.

### 3.2. Previous GA Experience

Overall, 52% and 5 % of PSN managed by private and public healthcare providers, respectively, underwent dental treatment prior to the referral for GA. A spectrum of various dental complaints/problems were reported by patients’ or patients’ legal guardians, with odontogenic pain being the most frequent (65%) in the private sector group and caries/cavities (54%) in PSN receiving care provided by public services (Table 5). There was a higher rate of previous GA episodes in patients receiving private care: 25% vs. 2.7%, respectively (Table 6).

### 3.3. A Range of Dental Treatment Carried out under GA

Evaluating the profile of procedures performed, those PSN treated by private providers underwent predominantly restorations (46%), as compared to those in the care of public services (33%). Extractions were the procedures most frequently carried out under GA (86% of patients overall) (Table 7). Professional mechanical plaque removal (supragingival and subgingival debridement) was a dominant procedure mainly in the public sector group (81%, *p* = 0.017) because of non-existing pre-GA dental treatment. Endodontic therapy, fixed prosthodontics (crowns), and biopsies of soft tissues were performed occasionally mainly by the private care providers (Table 8). Table 9 demonstrates the range of dental treatment in PSN diagnosed with specific medical conditions. Persons with intellectual disabilities, autistic spectrum disorder, and cerebral palsy required mostly extractions and restorations under GA.

## 4. Discussion

General anesthesia should be considered a viable option when other techniques (e.g., conscious sedation) fail to secure dental patients’ oral health with regard to safety and efficiency aspects [14]. Patients treated under GA presented with complex and various dental needs, enabling the justification of indication for GA as optimal for re-establishing their oral health status. Our study demonstrated that patients who received dental treatment under GA in public healthcare services tend to have more complex and severe medical conditions, as well as a higher risk of GA complications.

According to Blumer et al. [13], referrals made for dental GA imply that PNS are not compliant enough to receive dental care utilizing a routine approach or conscious sedation alternative. Consequently, examination under anesthesia (EUA) and the outcome of treatment under GA can be more predictable when compared with the provision of dental treatment in an outpatient setting [15]. However, the high cost of dental GA in a hospital setting can be the main reason affecting GA rate prevalence. Reportedly, the GA provision by independent/private providers may not be feasible due to prohibitive high cost [17]. However, due to growing demand for GA in special care dentistry, dental practitioners should be familiar with clinical indications, advantages, and risks involved in GA. Observational studies addressing the general profile of PSN treated under GA are deemed important from a public health point of view as they provide essential information for local authorities about the special needs of the population.

The proportion of male patients treated in the public sector was consistent with findings in other studies [18,19,20,21]. This phenomenon can be explained by the fact that some conditions are more prevalent in men (e.g., autism), severely disadvantaged groups of males, or generally poorer oral health. Other studies report a lack of cooperation of male patients diagnosed with ASD and unsuccessful dental care provision in the outpatient setting, justifying the need for GA referrals [18,21]. Undoubtedly, there is the issue of limited access of PSN to specialized, secondary care delivered by public healthcare providers, regardless of the economic status of the population. In addition, the vast proportion of individuals who declared themselves as black were unable to receive a private healthcare. Our results portrayed and rendered the considerable ethnic inequality, showing discrepancy in receiving comprehensive dental treatment within the private dental care sector.

When comparing private vs. public groups, the study elucidated that PSN and their guardians tend to seek dental care at a later stage in the lives of the former. Due to incoherent and conflicting public health policies for PSN, this cohort of patients lacks access to the most optimal care services considered proper to respond to their increasing dental care needs at their early stages of life. The private sector group was characterized by a greater discrepancy in patients’ ages, showing a wide variation concerning their oral health needs and consequently influencing their quality of life. It must be emphasized that PSN had substantially limited access to urgent and emergency care in the public sector [21]. Corroborating these findings, Ivancić Jokić et al. [22] reported that 63% of PSN had a history of multiple extractions of deciduous teeth after they reached three years of age. This indicates that children below five years of age require regular dental care, involving routine check-ups and prophylactic measures. In that study, 60% of participants were diagnosed with ASD and other intellectual disabilities. Similarly, Mallineni and You [14] showed that 60% of patients receiving dental treatment under GA had behavioral problems. In contrast, a study conducted in Mexico reported that cerebral palsy, intellectual disability, and Down syndrome condition are the most common diagnoses for patients who underwent GA in a hospital setting [23].

Regarding the routine dental care among participants prior to GA, more than half of the PSN made an attempt to be provided with dental care (52%) by private services. In contrast, only 5% of patients in the public sector sought dental care prior to GA. In the absence of financial constraints, the timely resolution of dental problems is a crucial factor, as pain was the most common factor in the private healthcare sector (65%), while dental caries was the most frequently reported issue in the public sector group (52%). Note that, respectively, Jockusch et al. [7] showed that 72% of patients who underwent dental treatment under GA complained of pain, which was due to delayed GA treatment associated with the existence of long waiting lists, lack of resources, and consequences of caries progression. Undoubtedly, identifying the patients’ major concerns can be challenging for caregivers, as PSN are often unable to recognize their dental problems [7].

Overall, only 17% of patients required a second session of dental treatment under GA. A recent study showed that 47% of patients diagnosed with autism experienced at least one procedure of GA prior to a subsequent course of treatment, occurring within two years in 24% of cases [12]. In our study group, considering treatment needs, 25% of the participants from the private sector group could be allocated and referred for dental care in public-funded dental centers. An important factor is attributed to a greater likelihood of post-GA monitoring of PSN in the private sector, allowing follow-ups and subsequent treatment for common dental problems. Some authors suggest that these patients require 2–6 months of follow-ups after GA to maintain their oral health and to avoid further dental interventions [24,25,26,27]. Based on our results, we believe that the adequacy of follow-up arrangement for PSN, depending on their needs, is currently a challenge for care providers, leading to the re-admission of patients. In order to reduce the high rate of oral diseases in PSN, the education of caregivers and patients must be improved and enhanced. This strategy is aiming to minimize the prevalence of oral diseases and GA interventions [28].

The existing evidence demonstrates that non-surgical multiple extractions are the most common procedures performed on PSN subjected to GA [25,26], which is supported by the findings of this study, in which 86% of the patients had at least one extraction completed. Multiple dental restorations were performed almost equally frequently by both groups of care providers, with the number of fillings being between one and four. Our findings are partially coherent with previous studies, while restorations are more common than extractions in younger patients [8,14]. In public funded health services, fewer restorations were performed (54%) compared to independent services (67%). This was likely attributed to more advanced carious lesions, ruling out the conservative approach.

The principal guidelines issued by the Royal College of Surgeons (UK) suggest extractions under GA for pre-cooperative, pediatric patients, including PSN, in case of high caries rate, symptomatic, severely compromised teeth, which are difficult to restore, and causing pain, and/or odontogenic infections or conditions that can lead to recurrent complications [29]. The clinical indications for GA in our study followed the RCS (UK) recommendations. Expectedly, the results of our study showed a low number of endodontic treatment procedures, crown/denture work, and biopsies performed under GA. There was no prosthetic treatment provided by public services, reflecting the limited restorative options in carefully selected cases. Jockush et al. [26] showed that only 2% of their PSN received endodontic treatment in single-rooted teeth, concluding that a radical GA approach is required for complex planning, thus reducing the risk of repeating a GA procedure in future.

### 4.1. Study Limitations

The limitations of this study, which might have had an impact on the analysis of the results, were incomplete patients’ records and lack of essential details about general anesthesia protocol and procedural aspects, which led to possessing insufficient information affecting data extraction. The detailed information on the type of GA depending on the severity of comorbidity was not the subject of our competence. This could potentially influence the consistency of the variables analyzed. Secondly, participant follow-ups can be compromised. Substantially more information was obtained from the private care providers, as the same GA team was involved in the follow-ups both at hospitals and outpatient clinics, whereas PSN receiving care in the public sector did not have follow-ups arranged and discharge letters to primary health units after the proposed treatment was completed. Moreover, the studied information comprised data collected from a single health service, thus restricting unbiased comparison. In addition, the sample size could impact the results and their interpretation. A multi-center, randomized, and structured study design with a sample size calculation is required to validate the obtained data. The slight variations in GA referrals criteria among healthcare services might also increase the risk of hidden bias.

### 4.2. Implications

We urge public health authorities and policy makers to support the access to multidisciplinary dental care for PSN, with enhanced training in dental sedation. Dental professionals require postgraduate structured training to cater to the need for dental treatment under GA, as well as to select the most appropriate treatment options for PSN, depending on individual risk factors, medical needs of patients, and ultimately on the level of cooperation. The use of non-pharmacological or pharmacological techniques available in the outpatient care setting as an alternative to GA reduces the GA referrals rate. The implementation of oral health education programs in PSN seems essential as a long-term preventive measure in maintaining stable and sound oral health, minimizing further risks attributed to GA. A wider promotion of campaigns devoted to special care dentistry and PSN is required to increase the awareness of the guardians, caregivers, and support workers regarding prophylactic measures preventing oral diseases development and progression in patients with high risk of oral health neglect. The financial aspect of GA arrangement in the private sector played a substantial role in healthcare service access. The access related to inequalities among populations may particularly affect the most disadvantages ones and underserved people.

## 5. Conclusions

Urgent and elective dental treatment under general anesthesia is an essential domain of the advanced dental management of middle-aged patients with special needs, mostly with diagnosed learning disabilities, provided by complementary public and private healthcare services. Underserved populations face barriers in access to advanced oral healthcare services, experiencing difficulties in addressing their oral health needs. The primary characteristics of PSN benefiting from GA may differ, and the range of dental procedures performed under GA varies, depending on the provider, with extractions and restorative treatment as the most frequently performed procedures. It can be noted that prompt and early dental intervention delivered in outpatient clinics mitigates the further need for complex dental treatment under general anesthesia. The healthcare commissioners and authorities are obliged to establish and support local dental services providing dental treatment for uncooperative patients with special needs.

## Figures and Tables

**Table 1:** Characteristics and demographics of study groups.

	Category	Private Provider		Public Provider		Total		*p*
		*n* = 63	%	*n* = 37	%	*n* = 100	%	
Gender *	**MaleFemale**	36	57.2	26	70.3	62	62	0.209
		27	42.8	11	29.7	38	38	
Ethnicity *	White	42	66.7	16	43.2	58	58	**0.035**
	Other	21	33.3	21	56.8	42	42	
Age **	Min–Max	6–80		10–57		6–80		**0.005**
	Mean (DM)	30 (17)		28 (11)		30 (15)		
	Median	29		25		26		
Municipality *	Rio de Janeiro	58	92	23	62	81	81	**<0.001**
	Other	5	8	14	38	19	19	

* Pearson’s chi-square test; ** ANOVA test.

**Table 2:** Sample distribution attributed to primary clinical diagnosis.

Clinical Diagnosis	Private Service		Public Service		Total	
	*n* = 63	%	*n* = 37	%	*n* = 100	%
**Autistisc Spectrum Disorder**	21	33.3	12	32.4	33	33
**Intellectual disability**	15	23.8	12	32.4	27	27
**Cerebral palsy**	7	11.1	3	8.1	10	10
**Down syndrome**	2	3.2	4	10.8	6	6
**Severe mental health condition**	3	4.7	2	5.4	5	5
**Lennox–Gastaut syndrome**	4	6.3	0	0	4	4
**Stroke**	2	3.2	0	0	2	2
**Dental phobia**	2	3.2	0	0	2	2
**Edwards syndrome**	1	1.6	1	2.7	2	2
**Amyotrophic lateral sclerosis**	1	1.6	0	0	1	1
**Phenylketonuria**	1	1.6	0	0	1	1
**Hydrocephalus**	1	1.6	0	0	1	1
**Coffin–Siris syndrome**	1	1.6	0	0	1	1
**Kabuki syndrome**	0	0	1	2.7	1	1
**Moebius syndrome**	1	1.6	0	0	1	1
**Pierre Robin syndrome**	1	1.6	0	0	1	1
**Prader–Willi syndrome**	0	0	1	2.7	1	1
**Attention deficit hyperactivity disorder**	0	0	1	2.7	1	1

**Table 3:** Sample distribution according to the medical condition categories and International Association of Disability and Oral Health classification.

Medical Categories	Private Provider		Public Provider		Total		
	*n* = 63	%	*n* = 37	%	*n* = 100	%	*p*
Behavioral and Psychiatric Disorder	26	41	15	40	41	41	0.942
Mental and Physical Disability	24	38	15	40	39	39	0.808
Congenital Anomalies/Syndromes	10	16	7	20	17	7	0.695
Chronic Systemic and/or Infectious Condition	3	5	0	0	3	3	0.177

Pearson’s chi-squared test.

**Table 4:** Profile of PSN considering pre-operative GA risk assessment.

Clinical Diagnosis	ASA I		ASA II		ASA III	
	Private	Public	Private	Public	Private	Public
**Autistisc Spectrum Disorder**	12 (57%)	4 (33%)	9 (43%)	8 (67%)	-	-
**Intellectual disability**	3 (20%)	1 (8%)	12 (80%)	10 (92%)	-	-
**Cerebral palsy**	-	-	7 (100%)	3 (100%)	-	-
**Down syndrome**	-	-	2 (100%)	3 (75%)	-	1 (25%)
**Severe mental health condition**	-	-	3 (100%)	2 (100%)	-	-
**Lennox–Gastaut syndrome**	-	-	4 (100%)	-	-	-
**Stroke**	-	-	1 (50%)	-	1 (50%)	-
**Dental phobia**	2 (100%)	-	-	-	-	-
**Edwards syndrome**	-	-	1 (100%)	-	-	1 (100%)
**Amyotrophic lateral sclerosis**	-	-	1 (100%)	-	-	-
**Phenylketonuria**	-	-	1 (100%)	-	-	-
**Hydrocephalus**	-	-	1 (100%)	-	-	-
**Coffin–Siris syndrome**	-	-	1 (100%)	-	-	-
**Kabuki syndrome**	-	-	-	-	-	1 (100%)
**Moebius syndrome**	-	-	1 (100%)	-	-	-
**Pierre Robin syndrome**	-	-	1 (100%)	-	-	-
**Prader–Willi syndrome**	-	-	-	1 (100%)	-	-
**Attention deficit hyperactivity disorder**	-	1 (100%)	-	-	-	-
**Total**	24 (38%)	8 (22%)	38 (60%)	26 (70%)	1 (2%)	3 (8%)

**Table 5:** Sample distribution based on main dental problems and complaints.

	Category	Private		Public		Total		*p*
		*n* = 63	%	*n* = 37	%	*n* = 100	%	
Main complaint *	Pain	41	65.1	13	35.1	54	54	**0.040**
	Caries/Cavity	16	25.4	20	54.1	36	36	
	Halitosis	4	6.3	3	8.1	7	7	
	Need of denture work	1	1.6	0	0	1	1	
	Oral lesion	1	1.6	1	2.7	2	2	

* Pearson’s chi-squared test.

**Table 6:** The attempts of dental treatment prior to general anesthesia and previous GA referrals.

	Category	Private		Public		Total		*p*
		*n* = 63	%	*n* = 37	%	*n* = 100	%	
Previous attempt *	No	31	49	27	73	58	58	**0.042**
	Behavioral management	19	30	4	11	23	23	
	Sedation	13	21	6	16	19	19	
**Previous general anesthesia**	Yes	16	25.4	1	2.7	17	17	**0.005**
	No	47	74.6	36	97.3	83	83	

* Pearson’s chi-squared test; attempt of dental procedures: either in the absence of or under minimal-to-moderate oral sedation or behavioral management technique alone.

**Table 7:** The prevalence of most common procedures performed under GA by public and private providers.

Variable *	Category	Private		Public		Total		*p*
		*n* = 63	%	*n* = 37	%	*n* = 100	%	
**Restorations**	No	21	33	17	46	38	38	0.444
	1–4	26	41	13	35	39	39	
	+4	16	26	7	19	23	23	
**Dental Extractions**	No	9	14	5	13	14	14	0.991
	1–4	35	56	21	57	56	56	
	+4	19	30	11	30	30	30	

* Pearson’s chi-squared test.

**Table 8:** The other dental procedures carried out under GA.

Variable *	Category	Private		Public		Total		*p*
		*n* = 63	%	*n* = 37	%	*n* = 100	%	
Periodontal non-surgical debridement (scaling)	Yes	36	57	30	81	66	66	**0.017**
	No	27	43	7	19	34	34	
Endodontics	Yes	9	14	2	5	11	11	0.429
	No	54	86	35	95	89	89	
Dental prosthesis (crown prosthesis)	Yes	3	5	0	0	3	3	0.294
	No	60	95	37	100	97	97	
Oral Biopsy	Yes	7	11	3	8	10	10	0.741
	No	56	89	34	92	90	90	

* Pearson’s chi-squared test.

**Table 9:** The performed dental procedures reflecting patients’ medical diagnosis.

	Restorations		Dental Extractions		Scaling		Endodontics		Dental Prosthesis		Oral Biopsy	
	Private	Public	Private	Public	Private	Public	Private	Public	Private	Public	Private	Public
**Autistisc Spectrum Disorder**	62	46	47	44	8	10	5	2	1	-	2	1
**Intellectual disability**	42	22	98	64	11	9	-	-	1	-	3	1
**Cerebral palsy**	21	2	34	9	6	3	3	-	1	-	-	-
**Down syndrome**	5	5	2	11	1	4	-	-	-	-	-	1
**Severe mental health condition**	-	2	29	6	1	2	-	-	-	-	1	-
**Lennox–Gastaut syndrome**	15	-	20	-	4	-	1	-	-	-	-	-
**Stroke**	1	-	4	-	2	-	-	-	-	-	-	-
**Dental phobia**	4	-	11	-	1	-	-	-	-	-	-	-
**Edwards syndrome**	-	-	5	4	-	1	-	-	-	-	-	-
**Amyotrophic lateral sclerosis**	3	-	13	-	1	-	-	-	-	-	-	-
**Phenylketonuria**	3	-	1	-	-	-	1	-	-	-	-	-
**Hydrocephalus**	5	-	3	-	-	-	2	-		-	-	-
**Coffin–Siris syndrome**	4	-	1	-	1	-	3	-	-	-	1	-
**Kabuki syndrome**	-	-	-	4	-	-	-	-	-	-	-	-
**Moebius syndrome**	-	-	4	-	-	-		-	-	-	-	-
**Pierre Robin syndrome**	9	-	2	-	-	-	-	-	-	-	-	-
**Prader–Willi syndrome**	-	-	-	2	-	1	-	-	-	-	-	-
**Attention deficit hyperactivity disorder**	-	-	-	1	-	-	-	-	-	-	-	-
**Total**	174	77	274	145	36	30	15	2	3	-	7	3

## Data Availability

Raw data are available on request.

## Abbreviations

ASA	American Society of Anesthesiologists
ASD	Autistic Spectrum Disorder
EUA	Examination Under Anesthesia
GA	General Anesthesia
IADH	International Association of Disability and Oral Health
PSN	Patients with Special Needs
RCA	Royal College of Surgeons
SPSS	Statistical Package for Social Science
UERJ	Universidade do Estado do Rio de Janeiro
